# Co-occurrence of hypertension and *diabetes mellitus* and simultaneous use of medications: Vigitel 2011 and 2021

**DOI:** 10.1590/1980-549720240060

**Published:** 2024-12-20

**Authors:** Veronica Batista Gomes Leitão, Priscila Maria Stolses Bergamo Francisco, Deborah Carvalho Malta, Karen Sarmento Costa

**Affiliations:** I Universidade Estadual de Campinas, Department of Collective Health – Campinas (SP), Brazil.; II Universidade Federal de Minas Gerais, School of Nursing – Belo Horizonte (MG), Brazil.

**Keywords:** Hypertension, Diabetes mellitus, Drug utilization, Cross-sectional studies, Behavioral risk factor surveillance system

## Abstract

**Objective:**

The risk of cardiovascular disease is about four times higher in patients with hypertension and *diabetes mellitus*, and its early detection is important to reduce mortality. The aim of this study was to estimate the co-occurrence of hypertension and *diabetes mellitus*, and the use of medications for the concomitant treatment of both diseases, in the population ≥50 years of age, according to health insurance/medical plan ownership, according to sociodemographic characteristics, and comparing the years 2011 and 2021.

**Methods:**

Using data from the Vigitel survey, crude and adjusted prevalence and prevalence ratios with 95% confidence intervals were estimated using Poisson regression with robust variance.

**Results:**

The prevalence of concurrent diagnosis of these conditions was 11.3% (95%CI 10.5–12.2) and 14.3% (95%CI 13.2–15.4) in 2011 and 2021, respectively. There was an increase of 32% among users with health insurance (11.1% in 2011 *vs*. 13.8% in 2021) and 46% among SUS users (11.4% in 2011 *vs*. 14.7% in 2021). In this period, there was an increase in concomitant drug use for the whole population, from 81.4% (95%CI 78.2–84.2) to 89.8% (95%CI 87.0–92.0). This increase can be attributed to the use of diabetes medications in both strata.

**Conclusion:**

This study showed that the simultaneous prevalence of hypertension and diabetes and the use of medication for these diseases were similar between SUS users and health insurance users, overcoming possible inequalities between the groups in the diagnosis and treatment of these conditions.

## INTRODUCTION

Demographic and epidemiological transitions have resulted in increased life expectancy at birth, influencing shifts in both mortality and morbidity rates in Brazil: as the population ages, mortality has become more prevalent among aged individuals, and a growing proportion of individuals are living with noncommunicable diseases (NCDs)^1^.

In response to this scenario, the Ministry of Health (MoH) launched the Strategic Action Plan to Combat Noncommunicable Diseases in Brazil, 2011–2022. This plan aimed to define and prioritize actions and investments to combat and curb the spread of NCDs over a 10-year period, following three key guidelines: a) surveillance, information, evaluation, and monitoring; b) health promotion; and c) comprehensive care^2^. The Surveillance System for Risk and Protection Factors for Chronic Diseases by Telephone Survey (*Sistema de Vigilância de Fatores de Risco e Proteção para Doenças Crônicas por Inquérito Telefônico* – Vigitel) is part of the MoH's Surveillance System for Risk Factors for NCDs, implemented in 2006 across all capitals of Brazil's 26 states and the Federal District (*Distrito Federal* – DF), with annual and continuous monitoring. Vigitel tracks the frequency and distribution of risk and protective factors for NCDs, contributing to the assessment of public policies. In 2011, data on the use of medications for treating hypertension and *diabetes mellitus* were collected, coinciding with the launch of the *Farmácia Popular/Saúde Não Tem Preço* Program, which began offering free medications for hypertension and diabetes to the population^2^.

In Brazil, between 2006 and 2021, the prevalence of hypertension among the adult population increased from 22.6 to 25.4%, while the proportion of adults with diabetes rose from 5.5 to 9.1%. In both genders, prevalence increased significantly with age and decreased with higher levels of education^3^. Data from the National Household Sample Survey (*Pesquisa Nacional por Amostra de Domicílios* – PNAD) for the years 1998, 2003, and 2008 revealed a rise in the simultaneous prevalence of these diseases. From the age of 50, significant increases were observed across the Brazilian regions, and among aged individuals (age ≥65 years), prevalence rates exceeding 15% were reported^4^. A study using 2012 data found that the simultaneous prevalence of these conditions among aged individuals (≥60 years) was 16.2%, with variations across Brazil's capitals^5^.

The association between hypertension and diabetes leads to an increased risk of kidney disease, coronary heart disease, stroke, and heart failure, as well as being linked to dyslipidemia, cardiac autonomic dysfunction, and a prothrombotic state^5^. It is important to note that health care, including access to medical consultations for diagnosis and monitoring, as well as access to medications, provides significant benefits in managing these conditions and improving the quality of life for individuals with NCDs^6^. Early detection of these diseases is crucial for implementing timely interventions — such as counseling on behavioral changes and the use of medications — aimed at reducing disease progression and mortality^7^.

In Brazil, both public and private health services are responsible for ensuring healthcare for the population. Established over 30 years ago, the Brazilian Unified Health System (*Sistema Único de Saúde* – SUS) provides free services nationwide at all levels of care^8,9^. Private health services are divided into two subsectors: supplementary health and classical liberal. The classical liberal subsector consists of independent private services, while the supplementary health sector includes services funded by health plans and insurance. This sector has steadily increased its market share: in 2000, approximately 31 million Brazilians were covered by health plans, a number that rose to 46 million in 2011 and 48.5 million in 2021 — about 1 in every 4 Brazilians^8–12^.

The population covered by health plans tends to be younger and healthier, primarily consisting of workers from public and private companies that offer these benefits to their employees. Individuals with private health plans or insurance report having better access to preventive services, as well as more frequent use of healthcare services compared to those without such coverage^9,12,13^. In the public sector, the Family Health Strategy (FHS) has led to significant improvements in the health of the Brazilian population by strengthening Primary Health Care (PHC). However, SUS continues to face underfunding, which may limit its ability to provide quality care and ensure full access to necessary health services for the population^9,12–15^.

The prevalence of individuals with two or more chronic diseases increases progressively with age and is associated with high mortality, reduced functional capacity, greater use of healthcare services, and poor medication adherence^16–18^. Therefore, the objective of this study was to estimate the simultaneous occurrence of hypertension and *diabetes mellitus*, as well as the use of medications for the concurrent treatment of both conditions among individuals aged 50 years old or older, based on health plan/medical insurance status and sociodemographic characteristics, comparing data from 2011 and 2021 — a decade after the launch of the Strategic Action Plan to Combat NCDs and the *Farmácia Popular/Saúde Não Tem Preço* Programs. A deeper understanding of the factors related to the treatment of these conditions is essential for the development of actions and interventions that address the needs of the population^19^.

## METHODS

This is a cross-sectional population-based study that utilized data from the 2011 and 2021 Vigitel Survey. The Vigitel sampling process employs a two-stage probability sampling method: initially, landlines are systematically selected in each city, with residential and active lines organized into subsamples. In the second stage, an adult resident (aged 18 years old or older) is randomly selected to respond to the questionnaire.

Over the years, there have been changes to the sample size. From 2006 to 2011, the minimum sample size was 2,000 individuals per city. Between 2012 and 2019, samples of 1,000 to 1,500 individuals were accepted in cities where landline telephone coverage was less than 40% of households and where the absolute number of households with a telephone was fewer than 50,000. In 2020 and 2021, the minimum sample size was set at 1,000 individuals per city. This sample size enables the estimation of the frequency of any risk or protective factor in the adult population with a 95% confidence level and a maximum error of four percentage points. Additionally, in 2021, the 27,093 interviews conducted were not distributed throughout the year, as had been the practice until 2019. Informed consent for participation in the survey was obtained orally during the telephone contact with the interviewees, and the Vigitel project received approval from the National Commission for Ethics in Research involving Human Beings of the Ministry of Health^20^.

This study utilized data from individuals aged ≥50 years who responded to the survey in 2011 (n=21,298) and 2021 (n=17,534). The analyses were stratified based on private health insurance status, categorizing respondents into users with health insurance and users of SUS. Data from both periods were combined into a single database to facilitate year-to-year comparisons. The distribution of the population across the different years was examined according to sociodemographic variables for individuals with and without health insurance/plan.

Having a health plan or health insurance was assessed through the question: "*Do you have a health plan or health insurance?*" (*yes, only one*; *yes, more than one*; *no*). The answers were subsequently classified as "*yes*" or "*no*." In this study, the variable was utilized as a proxy for use of SUS; thus, individuals without a health plan/health insurance at the time of the survey were regarded as exclusive users of SUS. This assumption is based on the premise that the proportion of individuals without a health plan who access private healthcare services through out-of-pocket payments — which are not included in the category of having a health plan/health insurance — is relatively small compared to the overall population.

A dichotomous variable was created to indicate the simultaneous occurrence of hypertension and *diabetes mellitus*, defined by individuals who responded positively to both of the following questions: "*Has a doctor ever told you that you have hypertension*?" and "*Has a doctor ever told you that you have diabetes*?" Subsequently, the prevalence and corresponding 95% confidence intervals were estimated according to sociodemographic variables: gender (male and female); skin color/race (white, black/brown); years of education (0–4, 5–8, 9–11, and 12 or more); and geographic region of residence (Central-West, Northeast, North, Southeast, and South).

Using the questions: "*Are you currently taking any medication to control hypertension?*" and "*Are you currently taking any medication to control diabetes?*" or "*Are you currently using insulin to control diabetes?*", another dichotomous variable was generated to assess the concurrent use of medications for the treatment of both chronic conditions. This variable considered individuals who responded positively to using oral medication for hypertension alongside either oral medication or insulin for diabetes. All estimates were calculated with 95% confidence intervals.

In the analyses, differences between proportions were assessed using Pearson's χ^2^ test (Rao-Scott), with a significance level of 5%. Subsequently, adjusted prevalence ratios (PR) and 95% confidence intervals were calculated through Poisson regression with robust variance. The prevalence rates were adjusted for age and education, taking into account changes in population composition over the study period. All analyses were conducted using Stata, version 16, within the "*svy*" module for complex sample data.

## RESULTS

**Figure 1:**
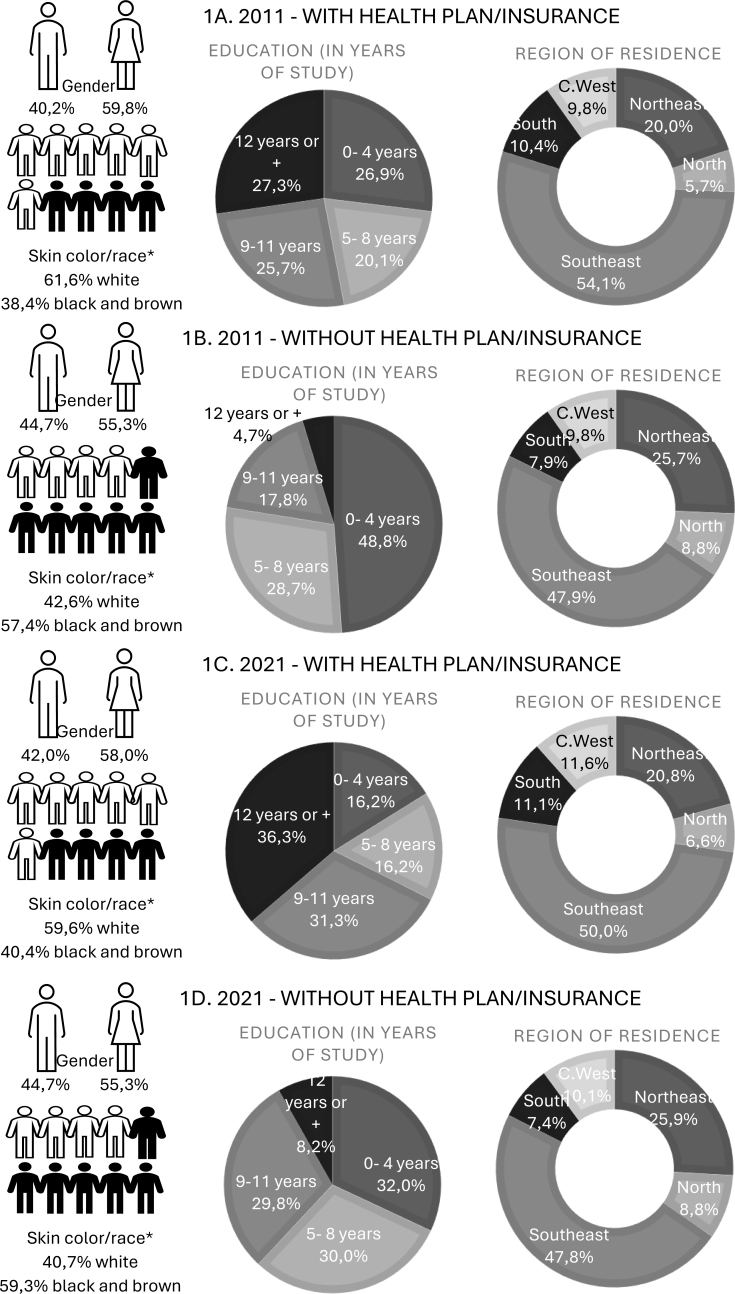
Infographic: Distribution of the adult population (aged 50 years old and older) according to health insurance ownership by sociodemographic variables. Vigitel, Brazil, 2011 and 2021.

From 2011 to 2021, the proportion of SUS users aged 50 years old or older increased from 50.3% (95%CI 48.8–51.6) to 54.9% (95%CI 53.2–56.6), while the proportion of health insurance users decreased from 49.7% (95%CI 48.3–51.1) to 45.1% (95%CI 43.4–46.8) of the adult population (data not shown in tables). Figure 1 illustrates that the population in both periods consisted predominantly of women, regardless of stratification. Among health insurance users, white individuals made up the majority, whereas among SUS users, black and brown individuals constituted the largest share. An increase in education levels was noted from 2011 to 2021 in both groups; however, disparities in education between individuals with and without health insurance remained significant.

The simultaneous prevalence of hypertension and *diabetes mellitus* among individuals aged ≥50 years old increased from 11.3% (95%CI 10.5–12.2) to 14.3% (95%CI 13.2–15.4) in the period. This rise occurred both among health insurance users, where prevalence increased from 11.1 to 13.8%, and among SUS users, with an increase from 11.4 in 2011 to 14.7% in 2021. A higher prevalence was observed among black/brown-skinned individuals with health insurance in 2011, while no significant difference in prevalence according to skin color was noted among SUS users. The prevalence was higher in the lower education strata across both years (Table 1).

**Table 1:** Simultaneous prevalence of hypertension and *diabetes mellitus* in the adult population (aged 50 years old and older), according to health plan/insurance ownership and demographic variables. Vigitel, Brazil, 2011 and 2021.

Year		2011 (n=21.298)			2021 (n=17.534)		
Characteristic and categories		Health plan/insurance users	SUS users	Total	Health plan/insurance users	SUS users	Total
		% (95%CI)	% (95%CI)	%(95%CI)	% (95%CI)	% (95%CI)	% (95%CI)
**Total**		11.1 (10.0–12.4)	11.4 (10.2–12.7)	11.3 (10.4–12.2)	13.82 (12.36–15.43)	14.66 (13.16–16.30)	14.27 (13.21–15.41)
Gender		p=0.092	p=0.055	p=0.009	p=0.378	p=0.585	p=0.340
	Male	9.8 (8.1–11.9)	10.0 (8.2–12.1)	9.9 (8.6–11.4)	13.0 (10.5–15.9)	14.2 (11.7–17.1)	13.6 (11.8–15.7)
	Female	12.0 (10.5–13.6)	12.6 (11.1–14.2)	12.4 (11.3–13.5)	14.4 (12.7–16.3)	15.1 (13.4–17.0)	14.8 (13.6–16.1)
Skin color*		p<0.001	p=0.522	p=0.002	p=0.009	p=0.567	p=0.030
	White	9.4 (8.0–10.9)	10.9 (9.0–13.0)	10.0 (8.9–11.2)	11.7 (9.9–13.7)	13.3 (11.1–15.9)	12.4 (11.0–14.0)
	Black/Brown	14.2 (12.1–16.7)	11.7 (10.1–13.5)	12.8 (11.5–14.2)	16.0 (13.5–18.9)	14.3 (12.2–16.6)	14.9 (13.3–16.7)
Education (years)†		p<0.001	p<0.001	p<0.001	p<0.001	p<0.001	p<0.001
	From 0 to 4	17.0 (13.9–20.5)	13.7 (11.8–15.9)	15.0 (13.3–16.8)	25.0 (20.6–30.1)	21.1 (18.0–24.5)	22.2 (19.7–25.0)
	From 5 to 8	12.9 (10.3–16.2)	10.1 (8.1–12.5)	11.3 (9.6–13.1)	16.0 (12.6–20.1)	14.42 (11.8–17.6)	14.9 (12.7–17.3)
	From 9 to 11	9.2 (7.7–11.1)	7.9 (6.2–10.0)	8.7 (7.5–10.1)	12.3 (9.7–15.4)	9.2 (7.2–11.6)	10.6 (9.0–12.5)
	12 or more	5.8 (4.7–7.1)	8.7 (5.3–13.9)	6.2 (5.1–7.5)	9.2 (7.3–11.5)	10.5 (6.6–16.1)	9.5 (7.7–11.6)
Region		p=0.514	p=0.467	p=0.954	p=0.013	p=0.394	p=0.015
	Central-West	11.7 (9.8–13.9)	11.5 (9.4–14.0)	11.6 (10.2–13.2)	10.7 (8.9–12.7)	13.9 (10.5–18.1)	12.3 (10.3–14.6)
	Northeast	11.7 (10.4–13.3)	11.4 (10.0–12.9)	11.5 (10.5–12.6)	14.9 (13.0–17.1)	14.9 (12.9–17.1)	14.9 (13.4–16.4)
	North	12.0 (9.7–14.8)	10.3 (8.5–12.3)	11.0 (9.6–12.6)	9.6 (7.2–12.7)	11.1 (8.8–14.0)	10.6 (8.8–12.7)
	Southeast	11.0 (9.1–13.3)	11.2 (9.0–13.8)	11.2 (9.7–12.9)	15.2 (12.6–18.2)	15.4 (12.7–18.5)	15.3 (13.4–17.4)
	South	9.4 (7.8–11.3)	14.0 (11.6–16.8)	11.4 (10.0–13.0)	11.3 (8.4–15.0)	14.6 (12.0–17.8)	12.8 (10.8–15.2)

SUS: Unified Health System; CI: confidence interval.

* data for Yellow and Indigenous individuals are not included, as the total number of observations in these categories was insufficient for reliable estimates;

† in years of education.

Among individuals with health insurance/plan, regional differences were observed in 2021, with lower prevalence rates in the Central-West and North regions compared to the Northeast (Table 1).

In the comparison between 2021 and 2011, an increase in the simultaneous prevalence of hypertension and diabetes was observed, with a prevalence ratio of 1.32 (95%CI 1.13–1.5) among health insurance users and 1.36 (95%CI 1.17–1.59) among SUS users. Stratified analyses revealed some differences: among individuals with health insurance/plan, the co-occurrence of these diseases increased in both white and black/brown populations; among SUS users, the prevalence increased only in black/brown populations. Educational level also showed variations, with an increase in prevalence among both higher and lower education levels for health insurance/plan users, while for SUS users, the increase was seen primarily in those with lower education levels (Table 2).

**Table 2:** Adjusted prevalence ratios for the simultaneous occurrence of hypertension and *diabetes mellitus* in adults (50 years old or older), according to health plan/insurance coverage, based on sociodemographic variables. Vigitel, Brazil, 2011 and 2021.

Characteristics		PR* 2021/2011 (95%CI)					
		Health plan/insurance users		SUS users		Total	
			p-value		p-value		p-value
Gender							
	Male	1.36 (1.03–1.81)	0.030	1.51 (1.13–2.02)	0.005	1.43 (1.17–1.75)	<0.001
	Female	1.30 (1.09–1.56)	0.004	1.27 (1.07–1.51)	0.006	1.27 (1.12–1.44)	<0.001
Skin color							
	White	1.28 (1.02–1.62)	0.032	1.23 (0.95–1.59)	0.114	1.26 (1.07–1.50)	0.007
	Black/Brown	1.26 (1.00–1.58)	0.047	1.37 (1.10–1.70)	0.005	1.28 (1.09–1.50)	0.002
Education (in years of study)†							
	From 0 to 4	1.44 (1.09–1.90)	0.010	1.45 (1.16–1.80)	0.001	1.42 (1.19–1.68)	<0.001
	From 5 to 8	1.10 (0.81–1.49)	0.529	1.42 (1.06–1.92)	0.020	1.29 (1.04–1.61)	0.022
	From 9 to 11	1.27 (0.93–1.72)	0.127	1.13 (0.79–1.60)	0.503	1.18 (0.94–1.48)	0.148
	12 or more	1.44 (1.05–1.99)	0.025	1.16 (0.59–2.26)	0.667	1.40 (1.05–1.86)	0.022
Region							
	Central-West	0.87 (0.67–1.11)	0.266	1.37 (0.97–1.95)	0.072	1.10 (0.87–1.40)	0.409
	Northeast	1.26 (1.04–1.53)	0.016	1.45 (1.19–1.77)	<0.001	1.36 (1.18–1.56)	<0.001
	North	0.86 (0.57–1.29)	0.475	1.25 (0.88-1.75)	0.206	1.07 (0.83–1.40)	0.584
	Southeast	1.50 (1.16–1.95)	0.002	1.39 (1.04–1.85)	0.024	1.42 (1.17–1.72)	<0.001
	South	1.31 (0.94–1.84)	0.113	1.07 (0.80–1.43)	0.637	1.19 (0.96–1.49)	0.116
Total		1.32 (1.13–1.54)	<0.001	1.36 (1.17–1.59)	<0.001	1.33 (1.19–1.48)	<0.001

PR: Prevalence ratios; SUS: Unified Health System; CI: confidence interval.

* adjusted for age and education;

† adjusted for age.

From 2011 to 2021, there was an increase in the concomitant use of medications for the treatment of hypertension and diabetes among the overall population (81.4 *vs*. 89.8%). When stratified by possession of a health plan, an increase in medication use was observed among SUS users (78.5 *vs*. 88.4%), while the percentage of use remained stable among those with a health plan/insurance. Furthermore, no significant differences in medication use were found between the groups when analyzed according to sociodemographic variables (Table 3).

**Table 3:** Prevalence of concurrent use of medications for the treatment of hypertension and *diabetes mellitus* in adults (50 years old or older) with a simultaneous diagnosis of these diseases, stratified by health plan/insurance coverage, based on sociodemographic variables. Vigitel, Brazil, 2011 and 2021.

Year		2011 (n=2.406)			2021 (n=2.807)		
		Health plan/insurance users	SUS users	Total	Health plan/insurance users	SUS users	Total
Characteristics		% (95%CI)	% (95%CI)	% (95%CI)	% (95%CI)	% (95%CI)	% (95%CI)
**Total**		**84.2 (79.1–88.2)**	**78.45 (74.1–82.3)**	**81.4 (78.2–84.2)**	**91.6 (87.9–94.2)**	**88.4 (84.1–91.6)**	**89.8 (87.0–92.0)**
Gender		p=0.814	p=0.112	p=0.441	p=0.744	p=0.414	p=0.361
	Male	83.4 (72.5–90.5)	82.7 (75.7–88.0)	83.0 (77.1–87.7)	90.9 (84.1–95.0)	86.6 (78.2–92.1)	88.4 (83.0–92.2)
	Female	84.6 (78.9–89.0)	75.8 (70.0–80.8)	80.4 (76.5–83.9)	92.0 (87.3–95.0)	89.8 (84.9–93.2)	90.8 (87.6–93.2)
Skin color		p = 0.485	p=0.051	p=0.092	p=0.889	p=0.155	p=0.357
	White	82.4 (74.5–88.3)	73.9 (65.5–80.8)	78.6 (73.2–83.2)	91.6 (85.9–95.2)	83.7 (74.0–90.3)	87.9 (82.5–91.8)
	Black/Brown	85.8 (78.2–91.0)	82.3 (77.3–86.4)	83.9 (79.8–87.3)	91.2 (84.6–95.1)	90.1 (84.3–93.9)	90.5 (86.4–93.5)
Education*		p=0.891	p=0.376	p=0.798	p=0.050	p=0.218	p=0.755
	From 0 to 4 years	85.7 (74.3–92.6)	78.2 (71.9–83.4)	81.4 (76.1–85.8)	88.0 (77.9–93.9)	90.6 (84.4–94.5)	89.8 (84.8–93.2)
	From 5 to 8 years	83.8 (73.5–90.7)	82.7 (75.3–88.3)	83.2 (77.3–87.9)	87.0 (76.4–93.2)	88.9 (79.5–94.3)	88.2 (81.5–92.7)
	From 9 to 11 years	82.3 (74.0–88.3)	74.7 (61.8–84.3)	79.4 (72.5–85.0)	96.4 (93.6–98.0)	87.0 (77.4–92.9)	92.0 (87.3–95.1)
	12 years or +	83.3 (75.2–89.2)	---†	79.8 (71.4–86.2)	93.9 (87.5–97.1)	73.0(44.1–90.2)	88.9 (78.9–94.5)
Region		p=0.989	p=0.722	p=0.774	p=0.355	p=0.448	p=0.266
	Central-West	84.2 (78.2–88.7)	77.2 (66.8–85.0)	80.6 (74.8–85.4)	89.2 (82.2–93.7)	91.1 (84.3–95.2)	90.3 (85.7–93.6)
	Northeast	84.8 (79.6–88.9)	76.8 (70.6–82.1)	80.3 (76.2–83.9)	92.4 (87.4–95.5)	90.4 (84.8–94.1)	91.2 (87.5–93.9)
	North	84.4 (76.0–90.3)	76.2 (66.8–83.6)	79.8 (73.5–85.0)	81.1(54.9–93.8)	83.9 (75.4–89.8)	83.1(73.2–89.8)
	Southeast	83.9 (74.1–90.5)	80.7 (72.0–87.2)	82.6 (76.4–87.4)	92.1 (85.6–95.8)	86.9 (78.3–92.4)	89.3 (84.0–92.9)
	South	84.1 (77.1–89.3)	75.4 (66.1–82.8)	79.5 (73.7–84.3)	94.0 (86.3–97.5)	91.9 (84.2–96.1)	92.9 (87.9–95.9)

SUS: Unified Health System; CI: confidence interval.

* in years of study;

† number of observations was insufficient for reliable estimates.

**Figure 2:**
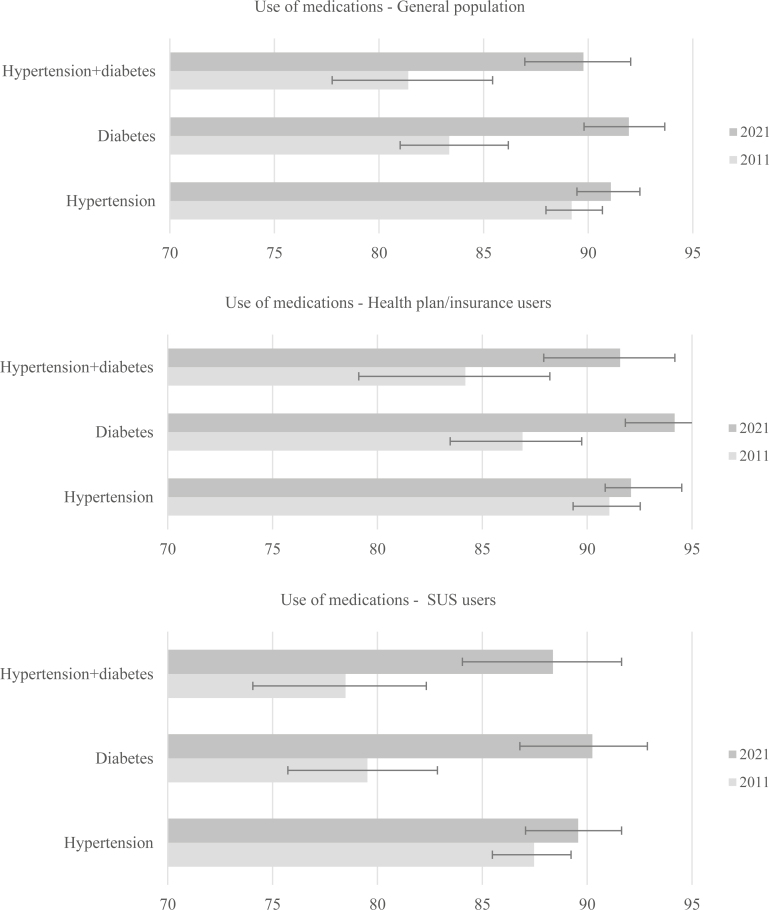
Prevalence of medication use for the treatment of hypertension, *diabetes mellitus*, and concurrent use for both diseases in adults (aged 50 years old and older) with a simultaneous diagnosis of hypertension and diabetes. Vigitel, Brazil, 2011 and 2021.

Regarding the prevalence of medication use for the treatment of hypertension and *diabetes mellitus* from a comparative perspective, the analysis not only considered the concomitant use of both medications but also verified the use of antihypertensives and antidiabetics separately. This approach aimed to better understand the changes that occurred over the period. The use of medications for diabetes treatment increased in both the general population (83.4 *vs*. 91.9%; p<0.001) and among those with health insurance (86.9 *vs*. 94.2%; p<0.001), as well as among SUS users, whose medication use rose from 79.5 to 90.3% (p<0.001). However, the use of antihypertensives remained stable in the general population (p=0.066), among those with health insurance (p=0.144), and among SUS users (p=0.172) (Figure 2).

SUS: Unified Health System.

## DISCUSSION

The results of this study demonstrated an increase in the simultaneous diagnosis of hypertension and *diabetes mellitus* among individuals aged 50 years or older, regardless of whether they had a health insurance/plan. Additionally, an increase in the use of medications for the concomitant treatment of both conditions was observed during the period. When stratified by possession of a health insurance plan and by therapeutic class, the observed increase can be attributed specifically to the use of medications for the treatment of diabetes in both groups.

The increase in diagnoses of hypertension and diabetes, as well as their co-occurrence during the study period, is attributed to rising life expectancy, along with the epidemiological and nutritional transitions leading to a rise in NCDs^21^. Obesity, a significant risk factor for cardiovascular diseases^22^, has been linked to increased consumption of ultra-processed foods at the expense of traditional Brazilian foods such as rice, beans, fruits, and vegetables, in addition to a more sedentary lifestyle. The simultaneous prevalence of an unhealthy diet and physical inactivity among the aged reached 32.1% in 2015^23^. Prior studies have established a positive association between abdominal obesity, hypertension, and an elevated risk of cardiovascular diseases in older adults. Moreover, an increase in visceral fat is also related to insulin resistance and an increased risk of type 2 *diabetes mellitus* ^23–25^.

The prevalence of hypertension is approximately twice as high in individuals with diabetes compared to those without the condition, and the risk of cardiovascular disease is roughly four times higher in patients with these comorbidities^5^. Both diseases significantly impact individuals and health systems, leading to an increased number of medications in use, particularly among aged individuals. They also result in restrictions on activities, with a notable degree of limitation in daily activities, as well as an increase in hospitalizations and demand for health services. This rising demand across various levels of health care results in substantial costs for SUS, necessitating the reorganization, qualification, and expansion of care services^5^.

The number of users with health insurance agreements has been increasing in Brazilian capitals: according to data from the Vigitel survey, the percentage of individuals with health insurance rose from 41.8% in 2008 to 55% in 2017^26,27^. However, the National Health Survey (*Pesquisa Nacional de Saúde* – PNS) did not indicate growth in this segment from 2013 to 2019: 27.9% (95%CI 27.1–28.8) and 28.5% (95%CI 27.8–29.2), respectively^8^. This discrepancy found by Vigitel^26,27^ suggests that health insurance coverage is higher in large urban centers, such as capitals. The economic crisis following 2015, coupled with rising unemployment, may explain the stability in the proportion of people with health insurance between 2013 and 2019^28,29^.

Furthermore, the population studied had a high proportion of individuals with low levels of education, a common characteristic among aged Brazilians^30^. This study highlights that low education levels were strongly associated with a 42% increase in the co-occurrence of hypertension and diabetes during the study period, irrespective of health insurance plans. Low educational attainment is an important social determinant that can hinder access to health promotion and care services^6^, while contributing to the accumulation of risk factors, such as poor diet; reduced physical activity; and greater use of tobacco and alcohol, among others^30^.

A study on the use of health services among individuals with and without health insurance revealed that those without health insurance/plan had a lower prevalence of service use, hospitalizations, and medical consultations, even when affected by certain NCDs^6^. Another study, comparing the performance of medical consultations and screening exams between exclusive users of the SUS and beneficiaries of supplementary health plans, also found lower frequencies among the first group^21^. This disparity may contribute to a lower diagnosis rate, including for NCDs like hypertension and diabetes.

The present study demonstrated an increase in the simultaneous diagnosis of hypertension and *diabetes mellitus* from 2011 to 2021, both among SUS users and those with health insurance. Despite the fact that individuals with health insurance generally have greater access to health services^6,8,21^, including medical consultations for comorbidities, no inequality in the prevalence of diagnosis was observed between public and private service users. This suggests that access to diagnosis for the selected conditions occurs at similar rates between users receiving drug treatment in both public and private healthcare settings, effectively overcoming potential inequalities in the treatment of these conditions. Similar findings were reported by the Vigitel survey in 2008 and 2017, which showed no significant differences in the prevalence of hypertension and diabetes between users of supplementary health services and the general population^15,16,31,32^. These results underscore the crucial role of SUS in reducing healthcare inequities and ensuring access to diagnosis and treatment^33^.

It is important to highlight the expansion of the FHS in Brazil, with coverage increasing from 4.4% in 1998 to 70% in 2017, particularly in municipalities in rural areas and metropolitan regions where populations of lower socioeconomic status reside^8^. This expansion, along with the growth of PHC teams, may have contributed to the increase in diagnoses of both hypertension and diabetes during the period from 2011 to 2021. However, in some regions, significant proportions of incomplete PHC teams have been reported, which can undermine the quality of care and services provided to users^34^.

The increase in the use of medications for treating hypertension and *diabetes mellitus* supports the findings of previous studies^31,32^. One study, which evaluated medication acquisition sources, showed a rise in the use of diabetes medications between 2012 and 2018; however, the proportion of free supply remained consistent over this period, with a shift from obtaining medications at SUS unit pharmacies to units of the *Farmácia Popular* Program^32^. In comparison to the results from the 2019 PNS, the proportions found were higher for the use of antihypertensive medications and similar for diabetes treatment^35^. It is important to note that this study focused on individuals aged ≥50 years. Regarding the concomitant use of antihypertensive and antidiabetic medications, the percentages were high, with nearly 90% of individuals with an indication for use adhering to treatment by 2021. However, it is significant to consider that approximately 188,000 Brazilians in this age group, who should be using these medications, are not. Understanding the factors related to non-adherence is critical, as pharmacological therapy is a key component in managing these health conditions effectively.

Among the limitations of the study, it is important to consider that the sample was restricted to individuals with landline telephones, which may have led to underrepresentation of the North and Northeast regions due to lower landline coverage in these areas. Although landline coverage has generally declined across the country, the use of weighting factors helped minimize differences between populations with and without a telephone^3^. For certain comparisons, particularly those involving skin color, the reduced sample size may have decreased the statistical power of the hypothesis tests^36^. Additionally, the sample was stratified based on health plan ownership, which includes various types of plans/insurance with differing coverages. Given that health insurance in Brazil operates supplementary to SUS, individuals with health insurance may access both services (public and private), especially for obtaining medications, potentially influencing the study's findings.

The present study demonstrated that the simultaneous prevalence of hypertension and diabetes and the use of medication for these diseases were similar between SUS users and health insurance users, overcoming possible inequalities between the groups in the diagnosis and treatment of these conditions.
