# Supplementary material for: Co-occurrence of hypertension and diabetes mellitus and simultaneous use of medications: Vigitel 2011 and 2021
Source: Rev Bras Epidemiol. 2024 Dec 20;27:e240060. doi: 10.1590/1980-549720240060 (PMC11654867; doi:10.1590/1980-549720240060)
Supplement: Translation

# Translation: Co-ocorrência de hipertensão arterial e diabetes mellitus e uso simultâneo de medicamentos: Vigitel 2011 e 2021

**DOI:** 10.1590/1980-549720240060.2

**Authors:** Veronica Batista Gomes Leitão, Priscila Maria Stolses Bergamo Francisco, Deborah Carvalho Malta, Karen Sarmento Costa

**Affiliations:** I Universidade Estadual de Campinas, Departamento de Saúde Coletiva – Campinas (SP), Brasil.; II Universidade Federal de Minas Gerais, Escola de Enfermagem – Belo Horizonte (MG), Brasil.

**Keywords:** Hipertensão, Diabetes mellitus, Uso de medicamentos, Estudos transversais, Sistema de vigilância de fator de risco comportamental

## Abstract

**Objetivo:**

O risco de doença cardiovascular é cerca de quatro vezes maior em pacientes com hipertensão arterial e diabetes mellitus, e sua detecção precoce é importante para diminuir a mortalidade. O objetivo deste estudo foi estimar a ocorrência simultânea de hipertensão arterial e diabetes mellitus, e o uso de medicamentos para tratamento concomitante de ambas as doenças, na população ≥50 anos, segundo posse de plano de saúde/convênio médico, de acordo com características sociodemográficas e comparando os anos de 2011 e 2021.

**Métodos:**

Utilizando dados do inquérito Vigitel, foram estimadas as prevalências e razões de prevalência brutas e ajustadas com intervalos de confiança de 95%, pela regressão de Poisson com variância robusta.

**Resultados:**

A prevalência de diagnóstico simultâneo dessas condições foi de 11,3% (IC95% 10,5–12,2) e de 14,3% (IC95% 13,2–15,4) em 2011 e 2021, respectivamente. Houve crescimento de 32% entre os usuários com plano de saúde (11,1% em 2011 vs. 13,8% em 2021) e 36% nos usuários do SUS (11,4% em 2011 vs. 14,7% em 2021). No período, houve aumento no uso concomitante de medicamentos para o conjunto da população, de 81,4% (IC95% 78,2–84,2) para 89,8% (IC95% 87,0–92,0). Esse aumento pode ser atribuído ao uso de medicamentos para diabetes, em ambos os estratos.

**Conclusão:**

O presente estudo mostrou que a prevalência simultânea de hipertensão e diabetes, assim como o uso de medicamentos para essas doenças, foram semelhantes entre usuários do Sistema Único de Saúde (SUS) e os usuários de planos de saúde/convênio, superando possíveis desigualdades entre os grupos no diagnóstico e tratamento dessas condições.

## INTRODUÇÃO

As transições demográfica e epidemiológica levaram a um aumento da expectativa de vida ao nascer e impactaram nas mudanças nas taxas de mortalidade e morbidade no Brasil: com o envelhecimento populacional, a mortalidade passou a predominar entre os mais velhos, e observam-se proporções crescentes de pessoas vivendo com doenças crônicas não transmissíveis (DCNT)^1^.

Ante esse cenário, o Ministério da Saúde (MS) lançou, em 2011, o Plano de Ações Estratégicas para o Enfrentamento das Doenças Crônicas Não Transmissíveis no Brasil, 2011–2022, visando definir e priorizar as ações e os investimentos para o país enfrentar e deter o avanço das DCNT em 10 anos, fundamentado em três diretrizes principais: a) vigilância, informação, avaliação e monitoramento; b) promoção da saúde; e c) cuidado integral^2^. O Sistema de Vigilância de Fatores de Risco e Proteção para Doenças Crônicas por Inquérito Telefônico (Vigitel) compõe o sistema de Vigilância de Fatores de Risco para DCNT do MS, implantado em 2006 em todas as capitais dos 26 estados brasileiros e no Distrito Federal (DF), com periodicidade anual e contínua. Monitora a frequência e distribuição de fatores de risco e proteção para DCNT, apoiando a avaliação das políticas públicas do país e, em 2011, foram colhidos dados sobre o uso de medicamentos para tratamento de hipertensão arterial e diabetes mellitus. Trata-se do ano de início do Programa Farmácia Popular/Saúde Não Tem Preço, que passou a ofertar medicamentos para hipertensão e diabetes gratuitamente à população^2^.

No Brasil, de 2006 a 2021, a prevalência de hipertensão na população adulta passou de 22,6 a 25,4%, e a frequência de adultos com diabetes aumentou de 5,5 para 9,1%. Em ambos os sexos, aumentou consideravelmente com a idade e diminuiu com o nível de escolaridade^3^. Dados da Pesquisa Nacional por Amostra de Domicílios (PNAD) de 1998, 2003 e 2008 mostraram elevação das prevalências simultâneas das doenças: desde os 50 anos de idade, observaram-se aumentos significativos nas regiões brasileiras; entre idosos brasileiros (idade ≥65 anos), encontraram-se prevalências acima de 15%^4^. Em estudo realizado com dados de 2012, a prevalência simultânea das doenças em idosos (≥ 60 anos) foi de 16,2%, com variação nas capitais brasileiras^5^.

A associação entre hipertensão e diabetes resulta em maior risco de doença renal, doença cardíaca coronariana, acidente vascular encefálico e insuficiência cardíaca, além de estar relacionada à dislipidemia, disfunção autonômica cardíaca e a estado pró-trombótico^5^. Deve-se considerar que a assistência à saúde, incluindo acesso a consultas médicas para diagnóstico e acompanhamento, além do acesso a medicamentos, resulta em benefícios na assistência e melhoria da qualidade de vida das pessoas com DCNT^6^. A detecção precoce dessas doenças é importante para que intervenções oportunas — como aconselhamento para mudança de fatores comportamentais e o uso de medicamentos — sejam realizadas, buscando-se reduzir o agravamento das condições e a mortalidade^7^.

No Brasil, coexistem serviços de saúde públicos e privados, responsáveis por garantir a assistência à saúde dos brasileiros. Criado há mais de 30 anos, o Sistema Único de Saúde (SUS), com abrangência nacional, oferece serviços gratuitos a toda população em todos os níveis de atenção^8,9^. Os serviços privados são divididos em dois subsetores: saúde suplementar e liberal clássico. O subsetor liberal clássico é composto por serviços particulares autônomos e a saúde suplementar, composta pelos serviços financiados pelos planos e seguros de saúde, que vêm ampliando sua participação no mercado: em 2000, cerca de 31 milhões de brasileiros eram beneficiários de planos de assistência médica; esse número passou para 46 milhões em 2011 e 48,5 milhões em 2021 — aproximadamente 1 em cada 4 brasileiros^8–12^.

A população atendida pelos planos de saúde é mais jovem e saudável, composta especialmente por trabalhadores de empresas públicas e privadas que oferecem tais benefícios a seus funcionários. Pessoas atendidas pelos planos privados/convênios médicos afirmam ter melhor acesso a serviços preventivos, além de maior uso dos serviços de saúde do que aquelas que não dispõem de tais planos^9,12,13^. Nos serviços públicos, a Estratégia de Saúde da Família (ESF) trouxe inegáveis avanços à saúde da população brasileira, com o fortalecimento da Atenção Primária a Saúde (APS). No entanto, o SUS segue subfinanciado, o que potencialmente compromete sua capacidade de assegurar a qualidade do cuidado e o acesso integral da população aos serviços de saúde de que necessita^9,12–15^.

O número de pessoas com duas ou mais doenças crônicas aumenta progressivamente com a idade e associa-se à alta mortalidade, redução de atividade funcional e ao aumento no uso de serviços, além da não adesão ao uso de medicamentos^16–18^. Assim, o objetivo do estudo foi estimar a ocorrência simultânea de hipertensão arterial e diabetes mellitus, e o uso de medicamentos para tratamento concomitante de ambas as doenças na população com 50 anos ou mais, segundo posse de plano de saúde/convênio médico, de acordo com características sociodemográficas e comparando os anos de 2011 e 2021 — transcorrida uma década do início do Plano de Ações Estratégicas para o Enfrentamento das DCNT e dos programas Farmácia Popular/Saúde Não Tem Preço. Compreender melhor aspectos relacionados ao tratamento dessas condições torna-se necessário para o desenvolvimento de ações e intervenções que vão ao encontro das necessidades da população^19^.

## MÉTODOS

Trata-se de estudo transversal de base populacional que usou dados do Inquérito Vigitel de 2011 e 2021. O processo de amostragem do Vigitel emprega amostra probabilística em dois estágios: primeiro o sorteio sistemático de linhas de telefone fixo em cada cidade, sendo que as linhas residenciais e ativas são organizadas em subamostras. No segundo estágio, é sorteado um morador adulto (≥18 anos) para responder ao questionário.

Ao longo de suas edições, houve algumas alterações quanto ao tamanho da amostra. De 2006 a 2011, o tamanho amostral mínimo foi de 2 mil indivíduos por cidade; e de 2012 a 2019, foram aceitas amostras de mil a 1.500 indivíduos em cidades em que a cobertura de telefonia fixa fosse inferior a 40% dos domicílios e o número absoluto de domicílios com telefone fosse inferior a 50 mil. Em 2020 e 2021, o tamanho amostral mínimo foi de mil indivíduos por cidade. Essa amostra permite estimar, com nível de confiança de 95% e erro máximo de quatro pontos percentuais, a frequência de qualquer fator de risco e proteção na população adulta. Adicionalmente a essa mudança, no ano de 2021, as 27.093 entrevistas realizadas não foram distribuídas ao longo de todo o ano, como ocorreu até 2019. O consentimento livre e esclarecido do inquérito foi obtido oralmente no momento do contato telefônico com os entrevistados e o projeto Vigitel foi aprovado pela Comissão Nacional de Ética em Pesquisa para Seres Humanos do Ministério da Saúde^20^.

Este estudo utilizou dados de indivíduos com idade ≥50 anos que responderam à pesquisa em 2011 (n=21.298) e em 2021 (n=17.534). As análises foram estratificadas segundo posse de plano médico privado de saúde em usuários com plano de saúde e usuários do SUS. Os dados de ambos os períodos foram agrupados em um único banco de dados, para permitir comparações entre os anos. Foi verificada a distribuição da população nos diferentes anos, segundo variáveis sociodemográficas, nos indivíduos com e sem plano de saúde/convênio.

A posse do plano de saúde/convênio médico foi verificada pela pergunta: *"O(a) senhor(a) tem plano de saúde ou convênio médico?"* (s*im, apenas um; sim, mais de um; não*), cujas respostas foram classificadas em "*sim*" e "*não*". Neste estudo, a variável foi utilizada como *proxy* para utilização do SUS, ou seja, os indivíduos que não tinham plano de saúde/convênio à época da pesquisa foram considerados usuários exclusivos do SUS, já que a proporção de pessoas sem plano de saúde que utiliza serviços de assistência médica particular por meio de desembolso direto — não incluída na categoria com plano de saúde/convênio — pode ser considerada pequena, comparativamente ao conjunto da população.

Foi criada uma variável dicotômica para a ocorrência simultânea de hipertensão arterial e diabetes mellitus, considerando os indivíduos que responderam positivamente às duas seguintes questões: "*Algum médico já lhe disse que o(a) Sr.(a) tem pressão alta*?" e, "*Algum médico já lhe disse que o(a) Sr.(a) tem diabetes*?". Em seguida, estimaram-se as prevalências e respectivos intervalos de confiança de 95%, segundo variáveis sociodemográficas: sexo (masculino e feminino); cor da pele/raça (branca, preta/parda); escolaridade em anos de estudo (0–4, 5–8, 9–11 e 12 ou mais); região geográfica de residência (Centro-Oeste, Nordeste, Norte, Sudeste e Sul).

Com as questões: "*Atualmente, o(a) Sr.(a) está tomando algum medicamento para controlar a pressão alta*?" e "*Atualmente, o(a) Sr.(a) está tomando algum comprimido para controlar o diabetes*?" ou "*Atualmente, o(a) Sr.(a) está usando insulina para controlar o diabetes*?", foi gerada outra variável dicotômica para verificar o uso concomitante de medicamentos no tratamento de ambas as condições crônicas, considerando os indivíduos que responderam positivamente sobre o uso de medicamento oral para tratamento de hipertensão, juntamente com o uso oral de medicamentos para diabetes ou o uso de insulina para controle desta doença. Todas as estimativas foram realizadas com intervalos de confiança de 95%.

Nas análises, as diferenças entre as proporções foram verificadas pelo teste χ^2^ de Pearson (Rao-Scott), considerando-se um nível de significância de 5%. Posteriormente, calcularam-se as razões de prevalência (RP) ajustadas e intervalos de confiança de 95%, por meio de regressão de Poisson com variância robusta. Ajustaram-se as prevalências segundo idade e escolaridade, considerando mudanças na composição populacional no período. Todas as análises foram realizadas no *Stata*, versão 16, no módulo "*svy*" para amostras complexas.

## RESULTADOS

De 2011 a 2021, a proporção de usuários do SUS com 50 anos ou mais de idade aumentou de 50,3% (IC95% 48,8–51,6) para 54,9% (IC95% 53,2–56,6), enquanto os usuários de planos de saúde representaram 49,7% (IC95% 48,3–51,1) e 45,1% (IC95% 43,4–46,8) da população adulta, respectivamente (dados não apresentados em tabela). Na Figura 1, observa-se que a população foi composta majoritariamente por mulheres, em ambos os períodos, independentemente da estratificação. Entre os usuários de plano de saúde, as pessoas de pele branca foram maioria; dos usuários do SUS, pretos e pardos representaram a maior parcela. Observou-se aumento da escolaridade de 2011 para 2021, em ambos os estratos. No entanto, as diferenças na escolaridade das pessoas com e sem plano de saúde permaneceram acentuadas.

**Figura 1:** Infográfico: Distribuição da população adulta (50 anos ou mais) de acordo com a posse de plano de saúde segundo variáveis sociodemográficas. Vigitel, Brasil, 2011 e 2021.

No que se refere à prevalência simultânea de hipertensão arterial e diabetes mellitus, para o conjunto dos indivíduos ≥50 anos de idade, houve aumento, passando de 11,3% (IC95% 10,5–12,2) para 14,3% (IC95% 13,2–15,4) no período. Esse aumento foi observado tanto entre os usuários de plano de saúde, de 11,1 para 13,8%, quanto nos usuários do SUS, passando de 11,4 em 2011 para 14,7% em 2021. A prevalência das doenças foi maior nas pessoas de pele preta/parda usuárias de plano de saúde/convênio em 2011, sem diferença segundo cor da pele nos usuários do SUS. Maiores prevalências foram observadas nos estratos inferiores de escolaridades, em ambos os anos (Tabela 1).

**Tabela 1:** Prevalência simultânea de hipertensão arterial e diabetes mellitus na população adulta (50 anos ou mais), de acordo com a posse de plano de saúde/convênio médico e segundo variáveis sociodemográficas. Vigitel, Brasil, 2011 e 2021.

Ano		2011 (n=21.298)			2021 (n=17.534)		
Variáveis e categorias		Usuários de plano de saúde/convênio	Usuários do SUS	Total	Usuários de plano de saúde/convênio	Usuários do SUS	Total
		% (IC95%)	% (IC95%)	%(IC95%)	% (IC95%)	% (IC95%)	% (IC95%)
**Total**		11,1 (10,0–12,4)	11,4 (10,2–12,7)	11,3 (10,4–12,2)	13,82 (12,36–15,43)	14,66 (13,16–16,30)	14,27 (13,21–15,41)
Sexo		p=0,092	p=0,055	p=0,009	p=0,378	p=0,585	p=0,340
	Masculino	9,8 (8,1–11,9)	10,0 (8,2–12,1)	9,9 (8,6–11,4)	13,0 (10,5–15,9)	14,2 (11,7–17,1)	13,6 (11,8–15,7)
	Feminino	12,0 (10,5–13,6)	12,6 (11,1–14,2)	12,4 (11,3–13,5)	14,4 (12,7–16,3)	15,1 (13,4–17,0)	14,8 (13,6–16,1)
Cor da pele*		p<0,001	p=0,522	p=0,002	p=0,009	p=0,567	p=0,030
	Branca	9,4 (8,0–10,9)	10,9 (9,0–13,0)	10,0 (8,9–11,2)	11,7 (9,9–13,7)	13,3 (11,1–15,9)	12,4 (11,0–14,0)
	Preta/parda	14,2 (12,1–16,7)	11,7 (10,1–13,5)	12,8 (11,5–14,2)	16,0 (13,5–18,9)	14,3 (12,2–16,6)	14,9 (13,3–16,7)
Escolaridade (anos)†		p<0,001	p<0,001	p<0,001	p<0,001	p<0,001	p<0,001
	De 0 a 4	17,0 (13,9–20,5)	13,7 (11,8–15,9)	15,0 (13,3–16,8)	25,0 (20,6–30,1)	21,1 (18,0–24,5)	22,2 (19,7–25,0)
	De 5 a 8	12,9 (10,3–16,2)	10,1 (8,1–12,5)	11,3 (9,6–13,1)	16,0 (12,6–20,1)	14,42 (11,8–17,6)	14,9 (12,7–17,3)
	De 9 a 11	9,2 (7,7–11,1)	7,9 (6,2–10,0)	8,7 (7,5–10,1)	12,3 (9,7–15,4)	9,2 (7,2–11,6)	10,6 (9,0–12,5)
	12 ou mais	5,8 (4,7–7,1)	8,7 (5,3–13,9)	6,2 (5,1–7,5)	9,2 (7,3–11,5)	10,5 (6,6–16,1)	9,5 (7,7–11,6)
Região		p=0,514	p=0,467	p=0,954	p=0,013	p=0,394	p=0,015
	Centro-Oeste	11,7 (9,8–13,9)	11,5 (9,4–14,0)	11,6 (10,2–13,2)	10,7 (8,9–12,7)	13,9 (10,5–18,1)	12,3 (10,3–14,6)
	Nordeste	11,7 (10,4–13,3)	11,4 (10,0–12,9)	11,5 (10,5–12,6)	14,9 (13,0–17,1)	14,9 (12,9–17,1)	14,9 (13,4–16,4)
	Norte	12,0 (9,7–14,8)	10,3 (8,5–12,3)	11,0 (9,6–12,6)	9,6 (7,2–12,7)	11,1 (8,8–14,0)	10,6 (8,8–12,7)
	Sudeste	11,0 (9,1–13,3)	11,2 (9,0–13,8)	11,2 (9,7–12,9)	15,2 (12,6–18,2)	15,4 (12,7–18,5)	15,3 (13,4–17,4)
	Sul	9,4 (7,8–11,3)	14,0 (11,6–16,8)	11,4 (10,0–13,0)	11,3 (8,4–15,0)	14,6 (12,0–17,8)	12,8 (10,8–15,2)

SUS: Sistema Único de Saúde; IC: intervalo de confiança.

* dados de amarelos e indígenas não incluídos, uma vez que o total de observações nestas categorias não foi suficiente para estimativas confiáveis;

† em anos de estudo.

Nos indivíduos com plano de saúde/convênio, houve diferenças segundo região de residência em 2021, com menores proporções nas regiões Centro-Oeste e Norte, em comparação ao Nordeste (Tabela 1).

Na comparação entre os anos de 2021 e 2011, observou-se aumento da prevalência simultânea das doenças, verificada por meio da razão de prevalência, de 1,32 (IC95% 1,13–1,5) nos usuários de plano de saúde e 1,36 (IC95% 1,17–1,59) nos usuários do SUS. Na avaliação estratificada, observaram-se algumas diferenças: naqueles indivíduos com plano de saúde/convênio, a co-ocorrência das doenças aumentou, tanto entre os brancos quanto entre pretos/pardos; já entre os usuários do SUS, a prevalência aumentou somente entre os pretos/pardos. Diferenças também foram verificadas quanto à escolaridade, com aumento da prevalência nas faixas superior e inferior naqueles com plano de saúde/convênio, e nos níveis inferiores de escolaridade nos que usavam o SUS (Tabela 2).

**Tabela 2:** Razões de prevalência ajustadas para a ocorrência simultânea de hipertensão arterial e diabetes *mellitus* em adultos (50 anos ou mais), de acordo com posse de plano de saúde/convênio médico, segundo variáveis sociodemográficas. Vigitel, Brasil, 2011 e 2021.

Variáveis		RP* 2021/2011 (IC95%)					
		Usuários de plano de saúde/convênio		Usuários do SUS		Total	
			Valor-p		Valor-p		Valor-p
Sexo							
	Masculino	1,36 (1,03–1,81)	0,030	1,51 (1,13–2,02)	0,005	1,43 (1,17–1,75)	<0,001
	Feminino	1,30 (1,09–1,56)	0,004	1,27 (1,07–1,51)	0,006	1,27 (1,12–1,44)	<0,001
Cor da pele							
	Branca	1,28 (1,02–1,62)	0,032	1,23 (0,95–1,59)	0,114	1,26 (1,07–1,50)	0,007
	Preta/parda	1,26 (1,00–1,58)	0,047	1,37 (1,10–1,70)	0,005	1,28 (1,09–1,50)	0,002
Escolaridade (em anos de estudo)†							
	De 0 a 4	1,44 (1,09–1,90)	0,010	1,45 (1,16–1,80)	0,001	1,42 (1,19–1,68)	<0,001
	De 5 a 8	1,10 (0,81–1,49)	0,529	1,42 (1,06–1,92)	0,020	1,29 (1,04–1,61)	0,022
	De 9 a 11	1,27 (0,93–1,72)	0,127	1,13 (0,79–1,60)	0,503	1,18 (0,94–1,48)	0,148
	12 ou mais	1,44 (1,05–1,99)	0,025	1,16 (0,59–2,26)	0,667	1,40 (1,05–1,86)	0,022
Região							
	Centro-Oeste	0,87 (0,67–1,11)	0,266	1,37 (0,97–1,95)	0,072	1,10 (0,87–1,40)	0,409
	Nordeste	1,26 (1,04–1,53)	0,016	1,45 (1,19–1,77)	<0,001	1,36 (1,18–1,56)	<0,001
	Norte	0,86 (0,57–1,29)	0,475	1,25 (0,88-1,75)	0,206	1,07 (0,83–1,40)	0,584
	Sudeste	1,50 (1,16–1,95)	0,002	1,39 (1,04–1,85)	0,024	1,42 (1,17–1,72)	<0,001
	Sul	1,31 (0,94–1,84)	0,113	1,07 (0,80–1,43)	0,637	1,19 (0,96–1,49)	0,116
Total		1,32 (1,13–1,54)	<0,001	1,36 (1,17–1,59)	<0,001	1,33 (1,19–1,48)	<0,001

RP: Razões de prevalência; SUS: Sistema Único de Saúde; IC: intervalo de confiança.

* ajustada segundo idade e escolaridade;

† ajustada segundo idade.

De 2011 a 2021, houve aumento no uso concomitante de medicamentos para tratamento de hipertensão e diabetes para o conjunto da população (81,4 *vs.* 89,8%). Quando estratificados por posse de plano de saúde/convênio, foi observado aumento do uso pelos usuários do SUS (78,5 *vs.* 88,4%). Naqueles com plano de saúde/convênio, o percentual de uso permaneceu estável. Ainda em relação aos medicamentos, não se observaram diferenças entre os estratos segundo as variáveis sociodemográficas (Tabela 3).

**Tabela 3:** Prevalência de uso concomitante de medicamentos para tratamento de hipertensão arterial e diabetes mellitus em adultos (50 anos ou mais) com diagnóstico simultâneo das doenças, estratificados por posse de plano de saúde/convênio médico, segundo variáveis sociodemográficas. Vigitel, Brasil, 2011 e 2021.

Ano		2011 (n=2.406)			2021 (n=2.807)		
		Usuários de plano de saúde/convênio	Usuários do SUS	Total	Usuários de plano de saúde/convênio	Usuários do SUS	Total
Variáveis		% (IC95%)	% (IC95%)	% (IC95%)	% (IC95%)	% (IC95%)	% (IC95%)
**Total**		**84,2 (79,1–88,2)**	**78,45 (74,1–82,3)**	**81,4 (78,2–84,2)**	**91,6 (87,9–94,2)**	**88,4 (84,1–91,6)**	**89,8 (87,0–92,0)**
Sexo		p=0,814	p=0,112	p=0,441	p=0,744	p=0,414	p=0,361
	Masculino	83,4 (72,5–90,5)	82,7 (75,7–88,0)	83,0 (77,1–87,7)	90,9 (84,1–95,0)	86,6 (78,2–92,1)	88,4 (83,0–92,2)
	Feminino	84,6 (78,9–89,0)	75,8 (70,0–80,8)	80,4 (76,5–83,9)	92,0 (87,3–95,0)	89,8 (84,9–93,2)	90,8 (87,6–93,2)
Cor da pele		p = 0,485	p=0,051	p=0,092	p=0,889	p=0,155	p=0,357
	Branca	82,4 (74,5–88,3)	73,9 (65,5–80,8)	78,6 (73,2–83,2)	91,6 (85,9–95,2)	83,7 (74,0–90,3)	87,9 (82,5–91,8)
	Preta/parda	85,8 (78,2–91,0)	82,3 (77,3–86,4)	83,9 (79,8–87,3)	91,2 (84,6–95,1)	90,1 (84,3–93,9)	90,5 (86,4–93,5)
Escolaridade*		p=0,891	p=0,376	p=0,798	p=0,050	p=0,218	p=0,755
	De 0 a 4 anos	85,7 (74,3–92,6)	78,2 (71,9–83,4)	81,4 (76,1–85,8)	88,0 (77,9–93,9)	90,6 (84,4–94,5)	89,8 (84,8–93,2)
	De 5 a 8 anos	83,8 (73,5–90,7)	82,7 (75,3–88,3)	83,2 (77,3–87,9)	87,0 (76,4–93,2)	88,9 (79,5–94,3)	88,2 (81,5–92,7)
	De 9 a 11 anos	82,3 (74,0–88,3)	74,7 (61,8–84,3)	79,4 (72,5–85,0)	96,4 (93,6–98,0)	87,0 (77,4–92,9)	92,0 (87,3–95,1)
	12 anos ou +	83,3 (75,2–89,2)	---†	79,8 (71,4–86,2)	93,9 (87,5–97,1)	73,0(44,1–90,2)	88,9 (78,9–94,5)
Região		p=0,989	p=0,722	p=0,774	p=0,355	p=0,448	p=0,266
	Centro-Oeste	84,2 (78,2–88,7)	77,2 (66,8–85,0)	80,6 (74,8–85,4)	89,2 (82,2–93,7)	91,1 (84,3–95,2)	90,3 (85,7–93,6)
	Nordeste	84,8 (79,6–88,9)	76,8 (70,6–82,1)	80,3 (76,2–83,9)	92,4 (87,4–95,5)	90,4 (84,8–94,1)	91,2 (87,5–93,9)
	Norte	84,4 (76,0–90,3)	76,2 (66,8–83,6)	79,8 (73,5–85,0)	81,1(54,9–93,8)	83,9 (75,4–89,8)	83,1(73,2–89,8)
	Sudeste	83,9 (74,1–90,5)	80,7 (72,0–87,2)	82,6 (76,4–87,4)	92,1 (85,6–95,8)	86,9 (78,3–92,4)	89,3 (84,0–92,9)
	Sul	84,1 (77,1–89,3)	75,4 (66,1–82,8)	79,5 (73,7–84,3)	94,0 (86,3–97,5)	91,9 (84,2–96,1)	92,9 (87,9–95,9)

SUS: Sistema Único de Saúde; IC: intervalo de confiança.

* em anos de estudo;

† número de observações não foi suficiente para estimativas confiáveis.

Quanto às prevalências de uso de medicamentos para tratamento de hipertensão arterial e diabetes mellitus numa perspectiva comparativa, além do uso concomitante de ambos os medicamentos, foi verificado separadamente o uso de anti-hipertensivos e antidiabéticos, para melhor compreender as mudanças ocorridas no período. O uso de medicamentos para tratamento de diabetes aumentou tanto na população geral (83,4 *vs*. 91,9%; p<0,001) quanto naquela com plano (86,9 *vs.* 94,2%; p<0,001), e na usuária do SUS, cujo crescimento observado foi de 79,5 para 90,3% (p<0,001). O uso para tratamento de hipertensão permaneceu estável na população geral (p=0,066), naqueles com plano (p=0,144) e na população atendida pelo SUS (p=0,172) (Figura 2).

**Figura 2:** Prevalência de uso de medicamentos para tratamento de hipertensão arterial, diabetes mellitus e uso concomitante para ambas as doenças em adultos (50 anos ou mais) com diagnóstico simultâneo de hipertensão e diabetes. Vigitel, Brasil, 2011 e 2021.

SUS: Sistema Único de Saúde.

## DISCUSSÃO

Os resultados deste estudo mostraram aumento de diagnóstico simultâneo de hipertensão arterial e diabetes mellitus na população com 50 anos ou mais, independentemente da posse de plano de saúde/convênio. Em relação ao uso de medicamentos para tratamento concomitante de ambas as doenças, também foi observado crescimento no período. Estratificando-se por posse de plano de saúde/convênio médico, e em seguida por classe terapêutica, o aumento pode ser atribuído ao uso de medicamentos para tratamento de diabetes, em ambos os estratos.

O aumento dos diagnósticos de hipertensão e diabetes, assim como de sua co-ocorrência no período estudado, relaciona-se com o aumento da expectativa de vida, além da transição epidemiológica, nutricional e do consequente aumento das DCNT^21^. A obesidade é um importante fator de risco para doenças cardiovasculares^22^ e é decorrente do aumento no consumo de alimentos ultraprocessados, em detrimento aos alimentos tradicionais brasileiros como arroz, feijão, frutas e hortaliças, bem como o aumento do sedentarismo. A prevalência simultânea de alimentação não saudável e inatividade física em idosos atingiu 32,1% em 2015^23^. Estudos anteriores estabeleceram associação positiva entre obesidade abdominal com hipertensão arterial e aumento do risco de doenças cardiovasculares em idosos. Além disso, o aumento da gordura visceral também está relacionado com resistência insulínica e elevado risco de diabetes melittus tipo 2^23–25^.

A prevalência de hipertensão é aproximadamente duas vezes maior nos indivíduos com diabetes, em comparação àqueles que não têm essa condição, e o risco de doença cardiovascular é cerca de quatro vezes mais elevado em pacientes com essas condições associadas^5^. Ambas as doenças têm elevado impacto tanto para os indivíduos quanto para os sistemas de saúde: aumento no número de medicamentos em uso, sobretudo entre os idosos; restrição de atividades com grau intenso/muito intenso de limitação de atividades diárias; e aumento nas internações e na procura por serviços. Esse aumento na procura, nos diversos níveis de atenção à saúde, resulta em elevados gastos ao SUS e lhe impõe a necessidade de reorganização, qualificação e ampliação do atendimento^5^.

Os usuários com plano de saúde/convênio vêm aumentando nas capitais brasileiras: segundo dados do inquérito Vigitel, em 2008, 41,8% dos indivíduos possuíam plano de saúde enquanto em 2017, esse percentual já era de 55% da população^26,27^. A Pesquisa Nacional de Saúde (PNS) não evidenciou crescimento deste seguimento de 2013 a 2019: 27,9% (IC95% 27,1–28,8) e 28,5% (IC95% 27,8–29,2), respectivamente^8^. Portanto, a maior proporção encontrada pelo Vigitel^26,27^ mostra que a cobertura de planos de saúde é maior nos grandes centros urbanos, como as capitais. A crise econômica após 2015 e o aumento do desemprego podem explicar a estabilidade na proporção de pessoas com planos de saúde entre 2013 e 2019^28,29^.

Ademais, a população estudada tem elevada proporção de pessoas com baixa escolaridade, característica comum entre idosos brasileiros^30^. Este estudo aponta a baixa escolaridade fortemente associada com a co-ocorrência de hipertensão e diabetes — aumento de 42% — no período estudado, independentemente de planos de saúde. Baixa escolaridade é um importante determinante social e pode interferir no pior acesso aos cuidados relacionados à promoção à saúde e assistência^6^, além de acúmulo de fatores de risco, como consumo de alimentação inadequada; menor prática de atividade física; maior uso de tabaco, álcool, dentre outros^30^.

Estudo sobre a utilização de serviços de saúde entre usuários com e sem planos de saúde mostrou que pessoas sem plano de saúde/convênio tiveram menor prevalência de uso de serviços, internação e consulta médica, mesmo na presença de alguma DCNT^6^. Outro estudo, que comparou a realização de consultas médicas e exames de rastreamento em usuários exclusivos do SUS com beneficiários da saúde suplementar, observou menores frequências no primeiro grupo^21^, o que pode influenciar em menor diagnóstico, incluindo as DCNT como hipertensão e diabetes.

O presente estudo mostrou crescimento, de 2011 a 2021, de diagnóstico simultâneo de hipertensão arterial e diabetes mellitus, tanto na população usuária do SUS quanto nas pessoas com plano de saúde/convênio. Portanto, embora os usuários que possuem plano de saúde/convênio tenham maior acesso aos serviços de saúde em geral^6,8,21^, incluindo consulta médica para essas comorbidades, não houve desigualdade na prevalência de diagnóstico entre usuários de serviços públicos e privados, ou seja, o acesso ao diagnóstico para as doenças selecionadas ocorre na mesma proporção entre os usuários em tratamento medicamentoso dos serviços públicos e serviços privados de saúde, superando possíveis desigualdades no tratamento dessas condições. Resultados semelhantes já haviam sido evidenciados pelo Vigitel em 2008 e 2017, ao analisar separadamente dados de usuários da saúde suplementar e da população geral, não evidenciando diferenças nos percentuais encontrados em ambos os grupos, tanto para hipertensão quanto para diabetes^15,16,31,32^. Esses achados destacam a importância do SUS na redução de iniquidades e no acesso ao diagnóstico e tratamento^33^.

Importante salientar a expansão da ESF no território brasileiro, com cobertura de 4,4% em 1998 e de 70% em 2017, principalmente em municípios do interior e das regiões metropolitanas em áreas onde residem populações com menor nível socioeconômico^8^, bem como das equipes de APS como fator que pode ter contribuído para o crescimento do diagnóstico de ambas as doenças no período de 2011 a 2021. No entanto, em algumas localidades, podem ser observadas consideráveis proporções de equipes incompletas, o que pode comprometer a assistência e o cuidado aos usuários^34^.

O aumento no uso de medicamentos para hipertensão arterial e diabetes mellitus corrobora os achados de estudos anteriores^31,32^. Estudo que avaliou as fontes de obtenção mostrou crescimento no uso de medicamentos para tratamento da diabetes de 2012 a 2018; no entanto, a proporção de fornecimento gratuito se manteve constante ao longo do período, com migração na obtenção nas farmácias das unidades do SUS para as unidades do Programa Farmácia Popular^32^. Em comparação aos resultados obtidos pela PNS 2019, as proporções encontradas foram superiores para uso de medicamentos para tratamento de hipertensão e semelhantes para o tratamento da diabetes^35^, contudo vale lembrar que este estudo utilizou dados de indivíduos ≥50 de idade. Em relação ao uso concomitante de anti-hipertensivos e antidiabéticos, embora os percentuais sejam altos, próximos a 90% dos usuários com indicação de uso em 2021, é relevante considerar que cerca de 188 mil brasileiros nesta faixa etária, com indicação de uso, não o fazem, tornando-se fundamental compreender os fatores relacionados a não adesão destes indivíduos, uma vez que para o controle adequado dessas condições de saúde, a terapia farmacológica é um dos componentes essenciais no processo de cuidado.

Entre as limitações do estudo, deve-se considerar que a composição da amostra se restringiu à população que possuía telefone fixo, o que pode ter diminuído a participação das regiões Norte e Nordeste do país, devido às menores taxas de cobertura. Em geral, a cobertura de telefone fixo tem declinado progressivamente no país, contudo, os fatores de ponderação minimizam as diferenças nas populações com e sem telefone^3^. Para algumas comparações realizadas, especificamente para a variável cor da pele, deve-se considerar que pode haver redução do poder do teste de hipótese devido ao menor tamanho da amostra nesta análise^36^. Também, a amostra foi estratificada segundo posse de plano de saúde. Existem vários tipos de planos/seguros de saúde, com diferentes coberturas. Uma vez que este mercado atua de forma suplementar, seus segurados podem acessar ambos os serviços (públicos e privados); portanto, entre os usuários com plano de saúde, pode haver indivíduos que utilizam também os serviços públicos e gratuitos ofertados pelo SUS, particularmente para a obtenção de medicamentos.

O presente estudo mostrou que a prevalência simultânea de hipertensão e diabetes, assim como o uso de medicamentos para essas doenças, foi semelhante entre usuários do SUS e usuários de planos de saúde/convênio, superando possíveis desigualdades entre os grupos no diagnóstico e tratamento dessas condições.
